# Specificity of functional network connectivity during the AD prodromal phase in mild cognitive impairment

**DOI:** 10.3389/fpsyt.2025.1722172

**Published:** 2026-01-16

**Authors:** Weiqing Li, Ze Feng, Bingyuan Chu, Qingqing Shang, Ming Yang, Xinlu Li, Hanxi Zhang, Xue Bai, Feng Wang

**Affiliations:** 1 Graduate School of Heilongjiang University of Chinese Medicine, Harbin, China; 2 Division of CT and MRI, The First Hospital Affiliated of Heilongjiang University of Traditional Chinese Medicine, Harbin, China

**Keywords:** amnestic mild cognitive impairment, brain networks, functional connectivity, non-amnestic mild cognitive impairment, resting-state functional magnetic resonance

## Abstract

**Background:**

Mild cognitive impairment (MCI) is a precursor state of Alzheimer’s disease (AD) and has attracted attention, but why amnestic mild cognitive impairment (aMCI) is more likely to progress to AD than non-amnestic mild cognitive impairment (naMCI) is unclear. The present study of aMCI compares differences in intra- and inter-network functional connectivity (FC) across multiple networks in naMCI and further correlates FC with cognitive assessment scores to assess their ability to predict AD progression.

**Methods:**

Resting-state functional magnetic resonance imaging (rs-fMRI) was performed in 30 naMCI and 40 aMCI cases, and 12 resting-state networks (RSNs) were identified by independent component analysis (ICA). Two-sample t-tests were performed to detect intra-network FC differences, and functional network connectivity (FNC) was calculated to compare inter-network FC differences. Subsequently, Pearson or Spearman correlation analyses were used to explore the correlation between altered FC and cognitive assessment scores.

**Results:**

The aMCI compared to the naMCI differed within the (Default mode network) DMN, (Dorsal attention network) DAN, (Sensorimotor system) SMN, and (Salience network) SN networks (corrected for FWEc, P< 0.05), and inter-network differences in DAN-DMN, DMN-SN, SN-SMN (corrected for FWEc, P<0.05).

**Conclusion:**

aMCI contrasts naMCI with widespread intra- and inter-static FNC differences, mainly involving the DMN, DAN, SMN, and SN. these network interactions provide a powerful method for assessing and predicting why aMCI is more likely to progress to AD, and contribute to our understanding of the neurological mechanisms underlying the pathological process of AD.

## 1 Introduction

Alzheimer’s disease (AD) profoundly disrupts older adults’ everyday functioning and social engagement. Early identification and intervention are therefore crucial for addressing cognitive decline. Mild cognitive impairment (MCI) represents an intermediate stage between normal aging and dementia, marked by modest deficits in domains such as memory, attention, language, and visuospatial abilities (1), People with MCI face a substantially higher risk of developing dementia—including AD—than cognitively healthy peers (2). Within MCI, a distinction has long been made between amnestic MCI (aMCI) and non-amnestic MCI (naMCI) subtypes. Among them, memory impairment is the most important defining feature of aMCI. Studies have shown that aMCI is more likely to progress to AD than naMCI (3), whereas naMCI is more likely to progress to non-AD dementias including, but not limited to, vascular dementia than aMCI.

Resting-state functional magnetic resonance imaging (rs-fMRI) acquires brain activity while participants rest, without presenting sensory inputs or requiring task performance, making it a popular tool for investigating aberrant neural activity and functional connectivity (FC) across clinical populations (4). FC quantifies the extent to which neural signals from different regions fluctuate together over time, providing a window into the functional organization of the brain (5). Consequently, an increasing number of investigators employ rs-fMRI to chart the temporal covariance of spontaneous neural activity, an approach that yields valuable information about the neural changes that occur during the prodromal phase of Alzheimer’s disease.

aMCI’s tendency to evolve into AD likely reflects disturbances at the network level — altered interactions among regions within a network and between multiple networks — rather than isolated dysfunction confined to a single brain locus. Traditional seed-based FC approaches depend on investigator-chosen regions of interest (ROIs) (6), which limits their capacity to capture broader inter-network dynamics. Independent component analysis (ICA), by contrast, is widely adopted to decompose resting-state data into spatially independent resting-state networks (RSNs), enabling identification of distributed functional systems (7). Building on ICA, functional network connectivity (FNC) characterizes moment-to-moment correlations between these RSNs and thereby provides a measure of inter-network communication patterns (8). Most aMCI studies have centered on comparisons with normal older adults or subjective cognitive decline or AD (9–11), and few studies have directly compared the combined neuropathology of aMCI and naMCI. Exploration of rsn and FNC may provide additional information to advance understanding of the underlying neural mechanisms that predispose aMCI to the development of AD.

Based on previous work and theoretical considerations, we hypothesized that aMCI and naMCI may have different FC patterns and systematically explored the differences between rsn as well as RSN in aMCI compared to naMCI. Temporal correlations of brain network activity were used to quantify their interactions and to estimate the extent to which between-group differences could predict the likelihood of developing AD.

## 2 Information and methods

### 2.1 Study population

Patients were recruited from the outpatient clinic of the Department of Acupuncture and Moxibustion of the First Hospital Affiliated to Heilongjiang University of Traditional Chinese Medicine from December 2024 to March 2025 and were first diagnosed with MCI. The diagnostic criteria for MCI were based on the Chinese expert consensus on assessment of cognitive impairment in the elderly〉 (12) and the Petersen Diagnostic Criteria (13), and also referenced to previous studies (14, 15). The results were summarized as follows. ①The scores on the Mini-Mental State Examination (MMSE) were ≤20 in the illiterate group, ≤23 in the elementary school group, and ≤26 in the middle school and above group. ②Montreal cognitive assessment (MoCA) scores were ≤13 points in the illiterate group, ≤19 points in the elementary school group, and ≤24 points in the secondary school and above group, and the age was 55–75 years old. Patients with MCI were recruited using this as a criterion. Compared with the age and education criteria, MCI patients with scores lower than -1.0 SD in the memory domain (sum of instantaneous and delayed memory) were categorized as aMCI, and the other part was categorized as naMCI. general information (age, gender, and education) and basic clinical information of the subjects were collected. Subjects voluntarily participated in this trial, understood and signed the informed consent form, and were approved by the Ethics Committee of the First Hospital Affiliated to Heilongjiang University of Traditional Chinese Medicine (Ethics No. HZYLLKY202500101).

Inclusion criteria of the observation group: (1) Meet the above diagnostic criteria of aMCI. (2) Excluding other diseases that can cause cognitive impairment; (3) Age 55-75; (4) Right-handedness; (5) Presence of one of the Aβ class biomarkers or neuronal damage markers detected by imaging and cerebrospinal fluid; (6) Not suffering from major psychiatric illnesses or serious heart, liver, kidney and other visceral dysfunctional diseases; (7) Not using drugs that affect cognitive ability for long periods of time or diseases that may affect cognitive performance; (8) not have pacemakers or metal fragments in the body that interfere with nuclear magnetic scanning.

Exclusion and exclusion criteria of the observation group: (1) subjects who had serious adverse events and could not proceed to the next step of the trial; (2) those who had sudden cerebrovascular diseases or craniocerebral trauma and other conditions in the course of the trial; (3) those who could not insist on completing the acupuncture treatment; (4) those who had incomplete clinical and imaging data; (5) those who had poor subjects’ adherence; and (6) those who had other uncontrolled factors or who withdrew from the experiment on their own.

### 2.2 Image data acquisition and pre-processing

FC analysis within FPN before and after needling was performed based on rs-fMRI. Observations were completed within 1 week before treatment and 1 week after all treatments were completed. This trial applied a Philips Ingenia 3.0T fully digital magnetic resonance scanner to acquire data. The gradient field strength was 40 mT/m, and a 16-channel parallel acquisition head coil (SENSE-NV-160) was applied, with 80 MHz high-frequency analog-to-digital converters per channel, direct digital sampling without analog filtering, and a gradient switching rate of 200 mT/m/ms. Detailed parameters of the structural image acquisition were Functional image acquisition was performed using a single-shot excitation planar echo imaging sequence (FFE single -shot EPI), the device acquisition parameters: repetition time (TR) = 2000ms, echo time (TE) = 30ms, matrix = 64 × 64, field of view (FOV) = 220mm × 220mm × 143mm, flip angle = 90°, number of scanned layers = 36, layer spacing = 1mm, layer thickness = 3mm, a total of 180 time points, and scanning time of 6min6s, and the scanning range covered the whole brain. Subjects were placed on the examination bed with their heads immobilized, close your eyes without sleeping or thinking about anything. Use foam padding to reduce involuntary head movements, and use earplugs to minimize noise impact on participants. All imaging data were post-processed by specialized personnel.

The acquired rs-fMRI image data were preprocessed by DPARSFA software based on MATLAB platform. First, the initial 10 time points are discarded to allow participants to adapt to the scanning environment. Subsequently, time correction is performed to correct for head motion. Data with head motion exceeding 2.0 mm translation or 2.0° rotation will be excluded. Secondly, the experimental data were aligned to the Montreal Neurological Institute standard space and resampled into 3 mm × 3 mm × 3 mm voxels for spatial normalization. Finally a Gaussian kernel was applied to spatially smooth the images for FWHM = 6 mm × 6 mm × 6 mm.

### 2.3 Neuropsychological assessment

Cognitive function was evaluated by a professionally trained neurologist in a quiet room free of external distractions. Overall cognitive function was screened using the Mini-Mental State Examination (MMSE) as well as the Montreal Cognitive Assessment (MoCA) scale, and quantitative memory scores were performed using the memory component of the MoCA.

### 2.4 ICA operations

The group ICA of fMRI toolbox software (http://icatb.sourceforge.net/) was used to extract RSNs. We applied ICA to derive functional networks in a data-driven manner, modeling the measured signals as linear mixtures of statistically independent sources. Dimensionality was reduced and components were estimated using the Fast-ICA framework implemented in GIFT v3.0b. Preprocessed fMRI datasets were loaded into GIFT, which automatically determined the model order (31 components in this dataset) and selected dimensionality-reduction parameters at both the individual and group levels. ICA decomposition was repeated 100 times using the infomax algorithm to ensure stable solutions, and spatial maps along with their associated time courses were reconstructed for each subject and at the group level. From the 31 resulting components, based on previous research (16–18), ten (corresponding to 6 canonical resting-state networks identified *a priori*) were retained for downstream analyses.

### 2.5 Internal RSN analysis

Of the 31 components produced by ICA, 10 (representing 6 resting-state networks) were chosen for further investigation. For each network, we first conducted a one-sample t-test to generate group-level z-maps; results were thresholded at P < 0.05 and corrected for multiple comparisons using family-wise error correction (FWEc) to produce statistical maps. Next, between-group differences were evaluated with two-sample t-tests applied to the RSN z-maps, restricting the analysis to voxels inside the combined mask and using an intergroup significance threshold of P < 0.01 with FWEc correction. Regions within each RSN that survived the two-sample comparison were extracted and carried forward for subsequent analyses.

### 2.6 Static FNC analysis between RSNs

Following ICA, the spatiotemporal dual regression method was employed to obtain the identified individual-level temporal processes of RSNs. Subsequently, FNC analysis was conducted to investigate the relationships among different RSN temporal processes. Temporal band-pass filtering (0.00–0.25 Hz) was first applied to each component time course to suppress slow drifts and high-frequency physiological artefacts. For every participant, pairwise correlations were then computed between all selected RSN time series. Pearson correlation coefficients produced a 10 × 10 FNC matrix for each subject, reflecting the strength of connectivity between networks. Group differences in FNC for each RSN pair were assessed using a general linear model, with multiple comparisons controlled by false discovery rate (FDR) at P < 0.05. Connections showing significant between-group differences were subsequently extracted and examined for associations with cognitive performance.

### 2.7 Correlation analysis

Imaging data were processed using Statistical Parametric Mapping software (Statistical Parametric Mapping, SPM12, http://www.fil.ion.ucl.ac.uk/spm) to calculate the correlation between FC in RSNs/FNC and cognitive function assessment in aMCI. For each RSN, the brain region that was significantly different in the two-sample t-test was selected as the ROI, and the coordinates of the ROI were extracted. Correlation analyses were then performed using the mean z-scores within the ROIs. In addition, for inter-network FC, significant differences between the three groups were detected at the 10-component level and their FNC coefficients were used to calculate the correlation with the assessment and cognitive scores.

### 2.8 Statistical analysis

For differences in demographic and clinical information between patients with aMCI and patients with naMCI, demographic and clinical characteristics of patients with aMCI and patients with naMCI were evaluated using independent t-tests for continuous variables and chi-square tests for proportions using the SPSS 25.0 package. p < 0.05 was taken as significant. Data normality was evaluated using the Shapiro-Wilk test, with a P value of > 0.05 indicating a normal distribution. We then used Cohen’s d to characterize the effect size (ES) of each clinical variable. For RSN and FNC analyses, two-sample t-tests were used for group comparisons between the aMCI and naMCI groups. Age, gender, and education level were used as covariates. Statistical values were P = 0.001 at the voxel level (uncorrected) and P < 0.05 at the CLUSTER level (FWEc corrected). Results were presented using xjView, BrainNet Viewer, and other software. Pearson or Spearman correlation was used to test the relationship between FC and neuropsychological test scores with statistical significance P < 0.05. During all analyses, voxel-level statistical analyses of RSNs were performed using SPM12, and FNC group comparisons and correlations were performed using MatLab functions (MatLab 2017b) with SPSS 25.0.

## 3 Results

### 3.1 Participants and clinical data

Demographic data are shown in **Table 1**.

**Table 1:** Demographic data.

Items	naMCI	aMCI	*P*-value
Demography			
Number	30	40	
Age(y)	64.90 ± 5.49	63.73 ± 5.36	0.372
Gender(M/F)	13:17	16:24	0.779
Education(y)	11.27 (9-12)	10.98 (9-12)	0.822
Neuropsychological			
MMSE scores	23.40 (22-25)	23.43 (21-26)	0.923
MoCA scores	21.23 (20-23)	20.28 (19-23)	0.448
Immediate memory			
1	n/a-	n/a	<0.001
2	n/a	3 (7.5)	
3	10 (33.3)	34 (85.0)	
4	15 (50.0)	3 (7.5)	
5	5 (16.7)	n/a	
Delayed memory			
1	n/a	1 (2.5)	<0.001
2	2 (6.7)	14 (35.0)	
3	24 (80.0)	25 (62.5)	
4	4 (13.3)	n/a	
5	n/a	n/a	

There were no statistically significant differences in demographics (age, gender, years of education) (p > 0.05) and no statistically significant differences in neuropsychological scores (MMSE, MoCA) between the two groups included (p < 0.05), but there was a statistically significant difference between the two groups in the memory domain scores (p < 0.05).

### 3.2 ICA and component selection

**Figure 1:**
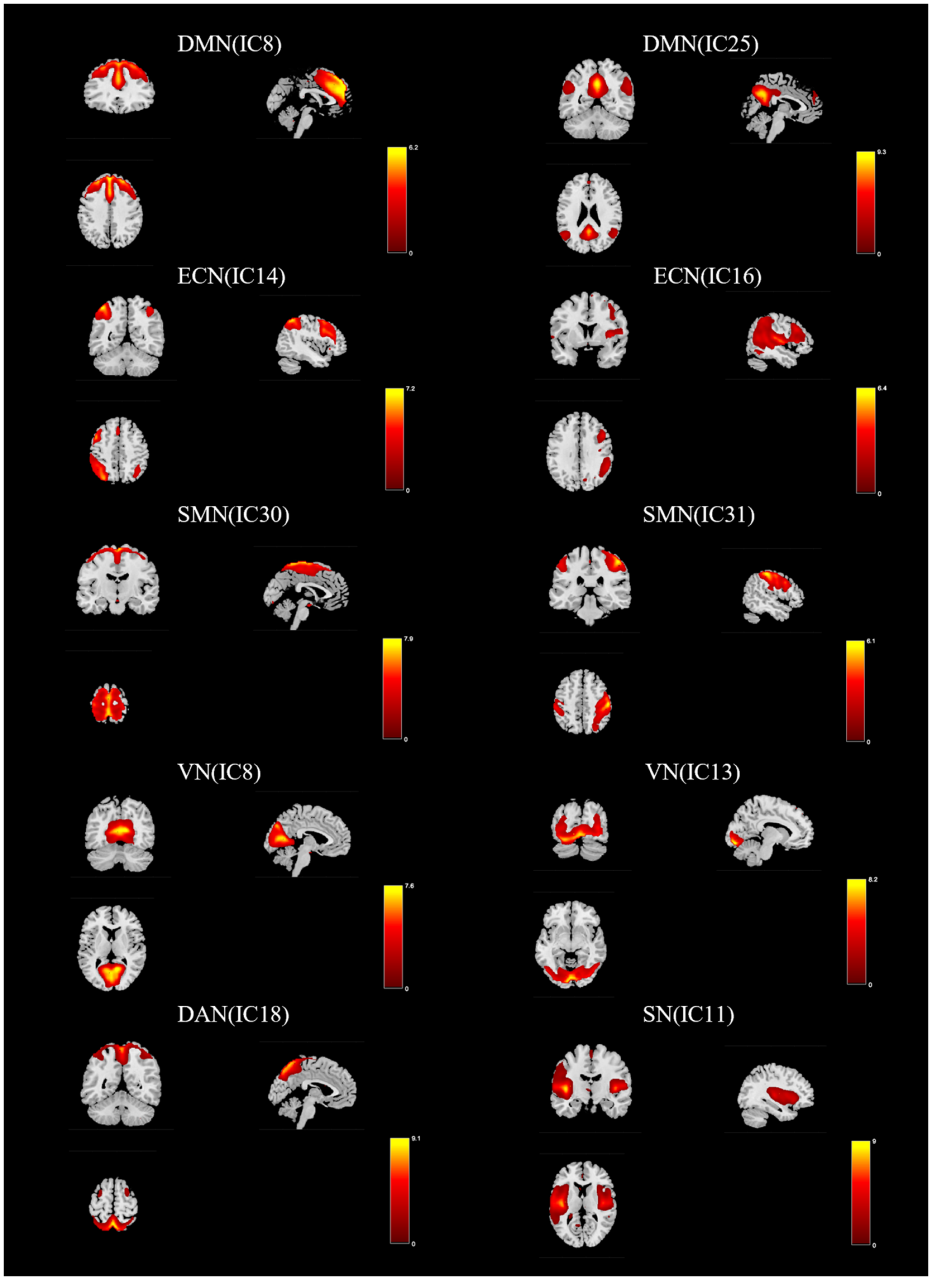
Functionally relevant RSN. Spatial maps of 10 independent components (ic) were selected for further analysis as RSN. DMN, default mode network; ECN, executive control network; SMN, sensorimotor network; VN, visual network; DAN, dorsal attentional network; SN, salient network.

In this study, ICA yielded 31 independent components, of which ten were chosen as RSNs for downstream analyses (see **Figure 1**). These components were grouped into seven canonical networks and labeled as follows:

1. Salience network (SN; IC11): Spatially dominated by the dorsal anterior cingulate cortex and anterior insula, with additional involvement of prefrontal regions.
2. Executive control network (ECN; IC14 + IC16): Composed of left and right frontoparietal subsystems. The left executive control network is centered on the left middle frontal gyrus, inferior and superior parietal lobules, and angular gyrus; the right executive control network shows a comparable topology on the right hemisphere.
3. Default mode network (DMN; IC8 + IC25): Encompasses typical DMN hubs including the posterior cingulate cortex, precuneus, inferior parietal lobule, bilateral angular gyri, and medial prefrontal cortex.
4. Dorsal attention network (DAN; IC18): Primarily involves the superior parietal lobule, frontal eye fields (prefrontal oculomotor area), and precentral gyrus.
5. Visual network (VN; IC3 + IC13): Covers early visual regions (including medial striate cortex and calcarine sulcus) and higher-order occipital areas such as the occipital pole, lateral occipital cortex, and fusiform gyrus.
6. Sensorimotor network (SMN; IC30 + IC31): Centered on primary motor and somatosensory cortices (precentral and postcentral gyri) and includes premotor and supplementary motor territories.

These network labels and component assignments were determined with reference to prior literature and the spatial topographies produced by our ICA decomposition.

### 3.3 Group FC differences within RSNs

**Figure 2:**
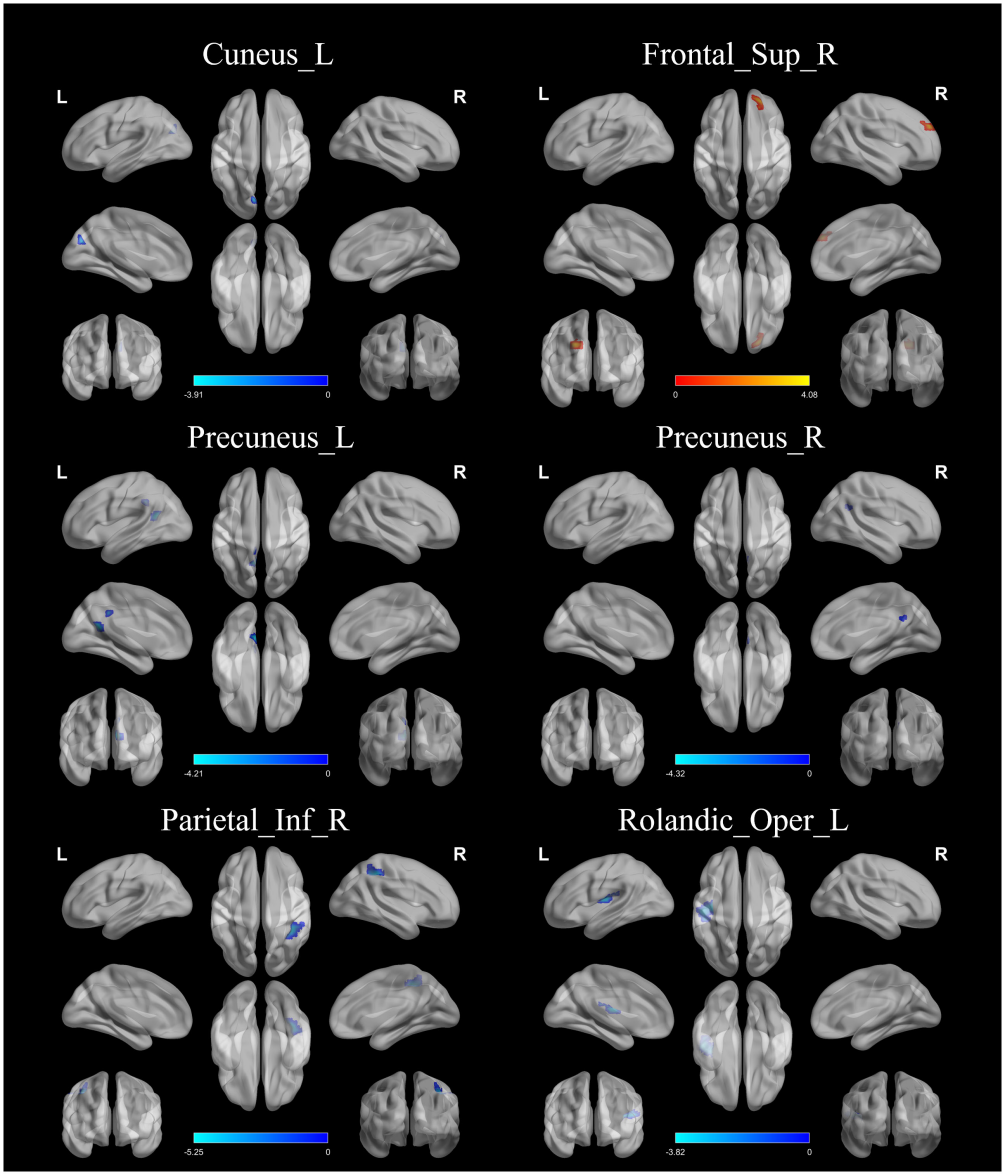
FC differences within RSNs. aMCI and naMCI groups differed significantly within the 4 RSNs. DAN, Precuneus_L; DMN, Precuneus_L, Precuneus_R and Frontal_Sup_R; SMN, Parietal_Inf_R; SN, Rolandic_Oper_L.

There were significant differences between the aMCI group and the naMCI group within the four RSNs, including DAN, DMN, SMN, and VN (**Table 2**, **Figure 2**). Lower FCs within DAN (Precuneus_L), DMN (Precuneus_L and Precuneus_R), SMN (Parietal_Inf_R) and VN (Rolandic_Oper_L) were observed in the aMCI group compared with the naMCI group. In addition, FC was higher in the DMN (Frontal_Sup_R) in the aMCI group compared to the naMCI group.

**Table 2:** Brain regions with significant differences in connectivity within RSNs between the aMCI and naMCI groups.

Brain networks	Brain regions	Brodmann	Peak *T* value	MNI		
				X	Y	Z
DAN	Cuneus_L	BA17	-3.9068	6	-72	33
DMN	Precuneus_L	BA31	-4.2063	-9	-54	18
	Precuneus_R	BA31	-4.3187	27	-63	30
	Frontal_Sup_R	BA9	4.0826	21	42	30
SMN	Parietal_Inf_R	BA40	-5.2477	36	-39	42
SN	Rolandic_Oper_L	BA13	-3.8172	-36	-12	15

Initial threshold for voxel level p<0.001 (uncorrected), cluster level threshold p<0.05 (FWEc-corrected). fc, functional connectivity. negative t-values indicate brain regions with reduced functional connectivity (FC) values, positive t-values indicate brain regions with enhanced functional connectivity (FC) values.

### 3.4 Differences between static FNC groups

**Figure 3:**
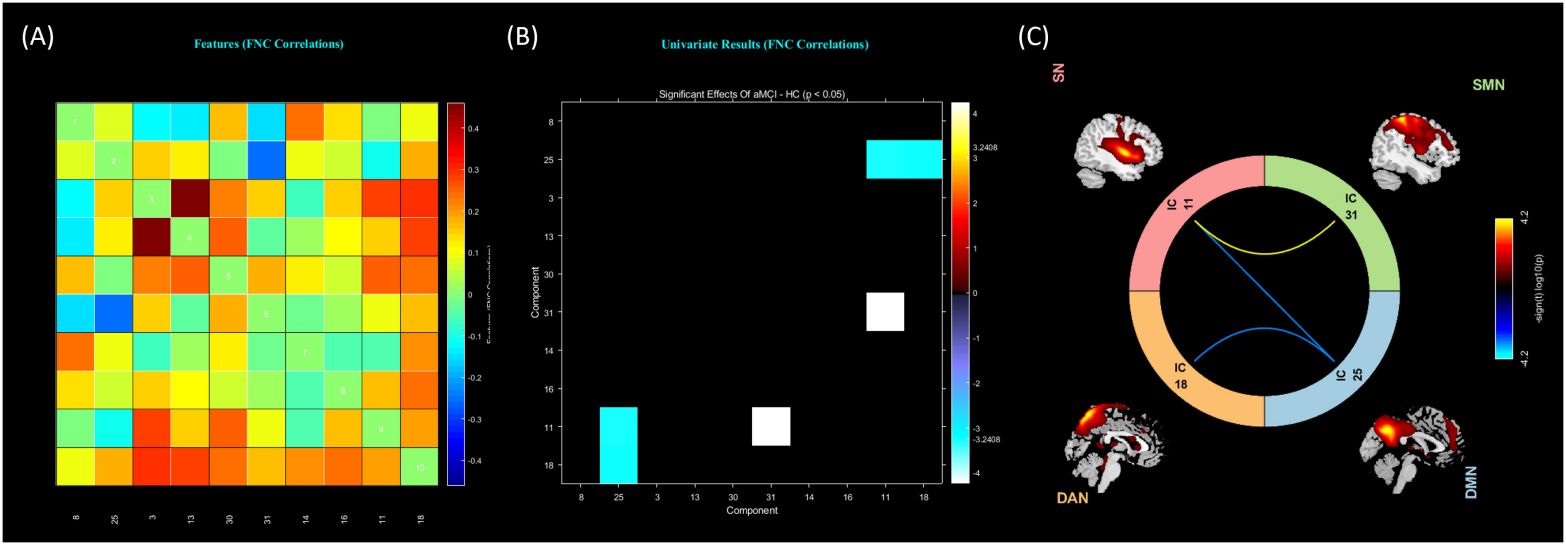
Static FNC intergroup differences. **(A)** FNC correlation matrix (averaged across subjects); **(B)** significant between-group effects between patients and controls; **(C)** 3 connections found to be significantly altered (P < 0.05).

In the FNC analysis, 3 connections were found to be significantly altered. The aMCI group had significantly fewer interactions in 2 RSN junctions (including the DMN-SN junction and the DMN-DAN junction) compared with the naMCI group. In addition, the FNC of SMN-SN interactions was increased in the aMCI group compared with the naMCI group (**Figure 3**).

## 4 Correlation analysis

**Figure 4:**
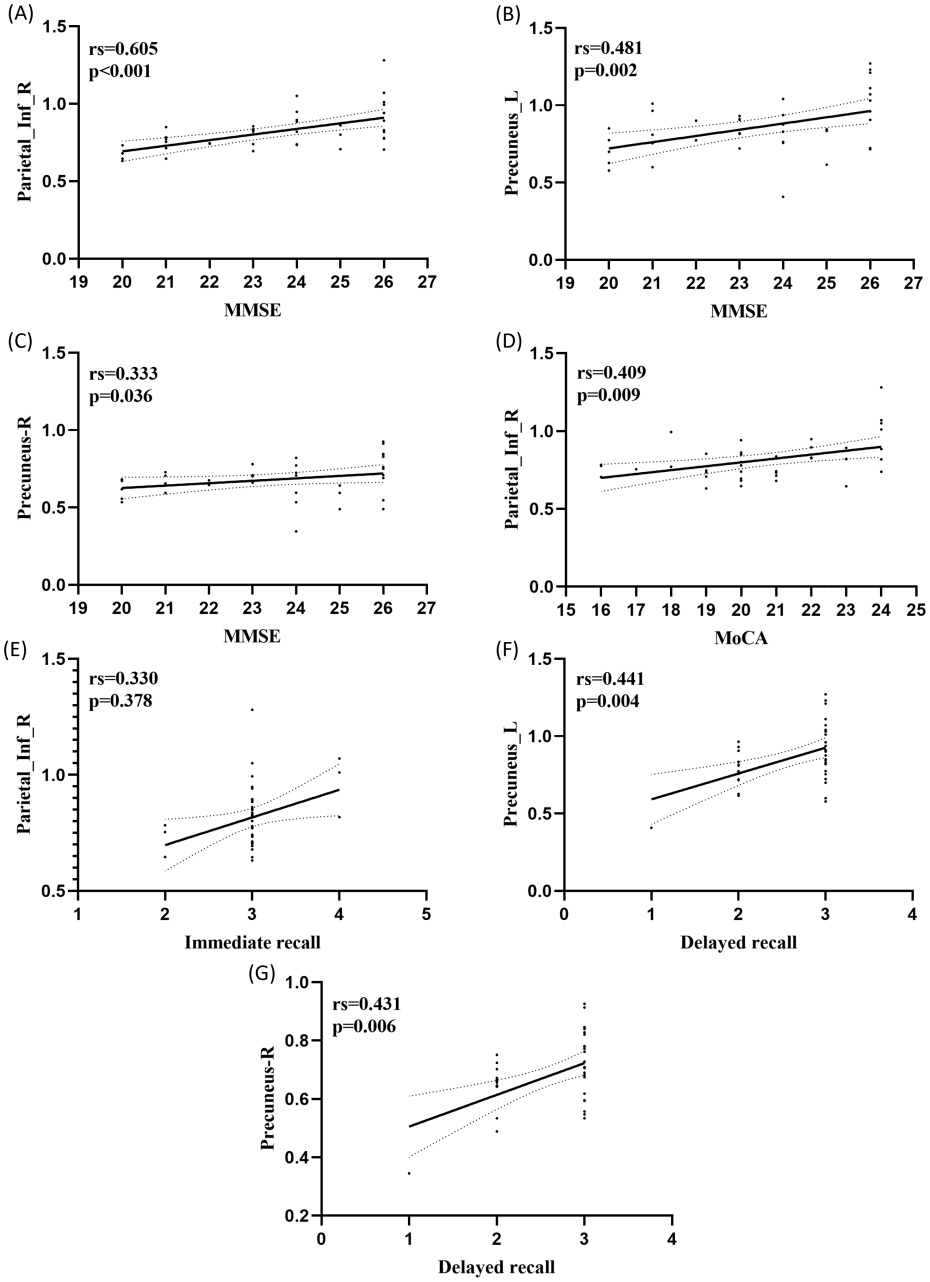
Correlation between mean z-scores and cognitive assessment scores for 5 ROIs in 5 RSNs of aMCI patients. **(A)** There was a significant positive correlation between Parietal_Inf_R within DMN and MMSE (rs = 0.605, p<0.001); **(B)** There was a significant positive correlation between Precuneus_L within DMN and MMSE (rs = 0.481, p = 0.002); **(C)** There was a significant positive correlation between Precuneus_R within DMN and MMSE (rs = 0.333, p = 0.036); **(D)** Parietal_Inf_R within DMN was significantly positively correlated with MoCA (rs = 0.605, P<0.001). **(E)** Parietal_Inf_R within DMN was significantly positively correlated with immediate memory (rs = 0.330, P = 0.378); **(F)** Precuneus_L within DMN was significantly positively correlated with delayed memory (rs = 0.441, P = 0.004); **(G)** Precuneus_R within DMN was significantly positively correlated with delayed memory (rs = 0.431, P = 0.006).

**Figure 5:**
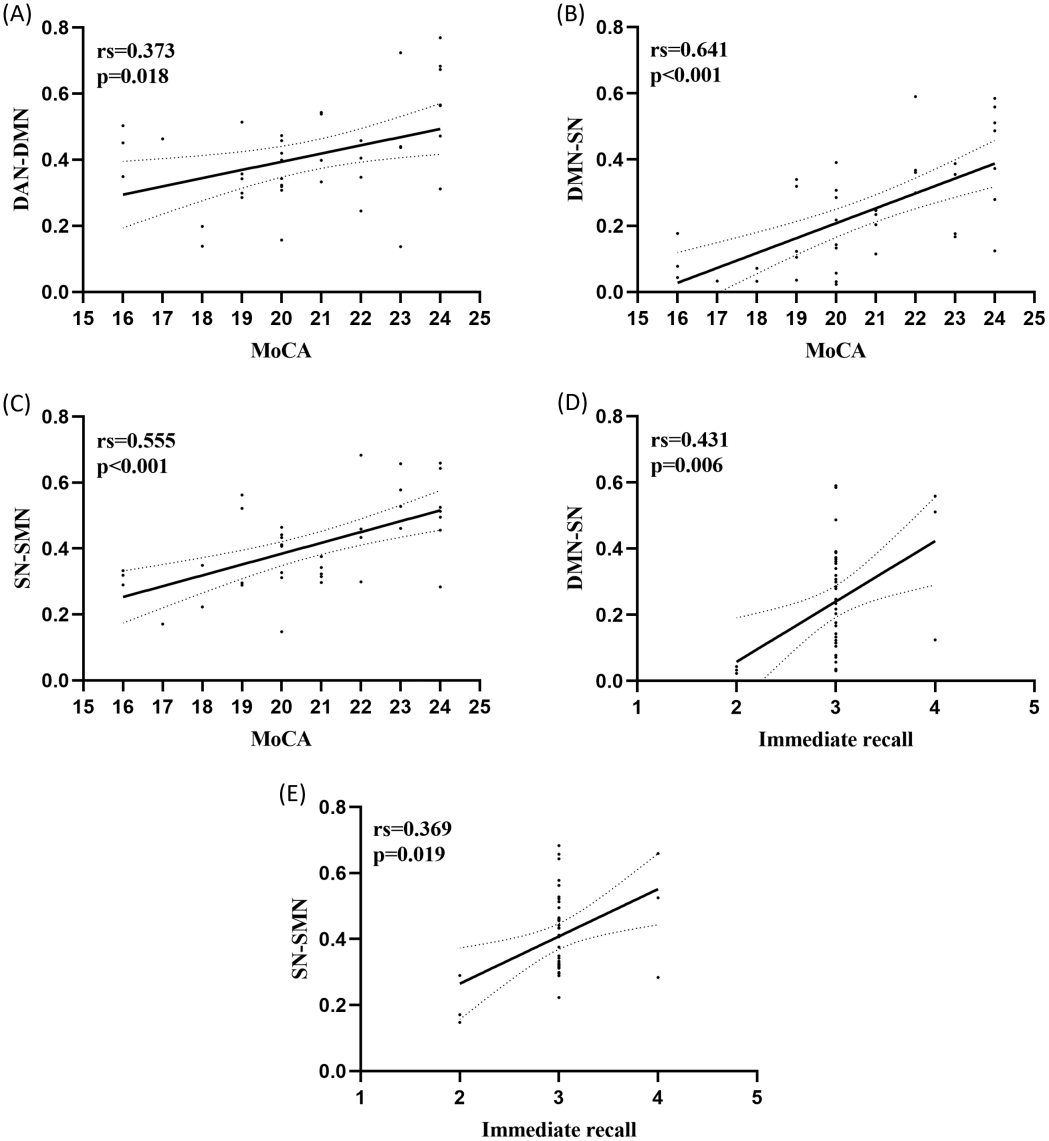
Correlation of differences between the 3 static FNC groups and cognitive assessment scores in aMCI patients. **(A)** DAN-DMN connections showed a significant positive correlation with MoCA (rs = 0.373, P = 0.018); **(B)** DMN-SN connections showed a significant positive correlation with MoCA (rs = 0.641, P<0.001); **(C)** SN-SMN connections showed a significant positive correlation with MoCA (rs = 0.555, P<0.001); **(D)** DMN -SN connections were significantly positively correlated with immediate memory (rs = 0.431, P = 0.006); **(E)** SN-SMN connections were significantly positively correlated with immediate memory (rs = 0.369, P = 0.019).

The mean z-scores of the five roi in the four RSNs were significantly positively correlated with the cognitive assessment scores, and there was a significant positive correlation between Parietal_Inf_R (IPL.R) and MMSE within the DMN (rs = 0.605, P<0.001),Precuneus_L (PCUN.L) and MMSE within the DMN (rs = 0.481, p = 0.002), and there was a significant positive correlation between Precuneus_R (PCUN.R) within DMN and MMSE (rs = 0.333, p = 0.036), in addition, IPL.R within DMN was significantly positively correlated with MoCA (rs = 0.605, p<0.001). There was a significant positive correlation between IPL.R within DMN and immediate memory (rs = 0.330, p=0.378), PCUN.L within DMN and delayed memory (rs = 0.441, p=0.004), and PCUN.R within DMN had a significant positive correlation with delayed memory (rs=0.431, p=0.006) (**Figures 4A–G**), Furthermore Correlation analysis of FNC coefficients (3 connections) with cognitive assessment scores revealed that DAN-DMN connections were significantly positively correlated with MoCA (rs = 0.373, p=0.018), DMN-SN connections were significantly positively correlated with MoCA (rs = 0.641, p<0.001), and SN-SMN connections were significantly positively correlated with MoCA (rs = 0.555, P<0.001). SN-SMN was significantly positively correlated with immediate recall (rs = 0.431, P = 0.006), and SN-SMN was significantly positively correlated with immediate recall (rs = 0.369, P = 0.019) (**Figures 5A–E**).

## 5 Discussion

To the best of our knowledge, this study is the first to use ICA method to detect intra- and inter-network FC and its relationship with cognitive functioning in aMCI patients compared to naMCI, and thus to investigate the mechanism why aMCI is more likely to progress to AD. In this study, different FCs were observed in several cognitive-related networks in aMCI patients compared to naMCI patients, i.e., DAN, DMN, SMN, and SN. Meanwhile, the FNC analysis revealed that differences in inter-network FCs among the three functionally connected networks appeared in aMCI patients, i.e., DAN-DMN was relatively lower versus DMN-SN, and SN-SMN was relatively higher.

The analysis of FC within the rsn focuses on the interaction of multiple brain regions in a certain spatial pattern. In this study, aMCI patients observed relatively low FC in Cuneus_L (CUN.L) in the DAN. the DAN is a key neural system in the human brain for purposeful and selective attention, which is essential for complex cognitive activities and information processing. disruption of the DAN is also a cause of memory impairment (19). And in DAN, the severity of FC disconnection increases with the progression of AD disease (20). CUN.L as a part of DAN plays an important role in emotional processing, control of cognitive functioning and behavior, its impairment is consistent with previous studies (21), and Cuneus impairment has also been found to be significantly associated with higher risk of AD (22).

The DMN, as the most widely studied brain network in the AD process, is closely related to, among others, memory in higher cognition (23, 24). As a major node in the DMN, Precuneus plays a central role in cognitive control and executive functions (25), as argued by the correlation between bilateral Precuneus and MMSE in this paper, in addition to its significant association with delayed memory, which has been widely confirmed (26, 27). A growing body of evidence reveals a central role for Precuneus in AD (28, 29). In particular, it is now relatively clear that functional connectivity abnormalities in the Precuneus are central to the development of AD as pathology accumulates, and that even FC abnormalities may precede the onset of symptoms (30), which is in line with the theory that aMCI is prone to progress to AD.

Frontal_Sup_R (SFGdor.R) has been shown to be significantly associated with both AD and dementia with Lewy bodies (31, 32), and dementia with Lewy bodies is one of the main progression targets of naMCI. And according to a previous study the right prefrontal lobe where SFGdor.R is located in naMCI would be more damaged than in aMCI (33), which inferred that the FC of SFGdor.R is stronger in aMCI, which is consistent with our study.

The SMN consists primarily of visual, auditory, and sensorimotor cortex. Previous studies have claimed that changes in sensory and motor function may precede cognitive symptoms of AD and may increase the risk of AD (34). In addition, the SMN has been suggested to be a commonly impaired network in the prodromal stages of AD (35). The Inferior parietal gyrus is a convergence zone for various brain networks, and is critical for the realization of hierarchically distinct neuroprocessing structures critical to cognition. This includes low-level processes, such as spatial attention, as well as significantly more complex high-level processes, such as semantic memory and social exchange patterns (36), and its importance with cognition is also reflected by the significant positive correlation of IPL.R with the MMSE and the MoCA. Cheng et al. (37) found that IPL.R was associated with poorer performance in Immediate recollection, which significantly identified aMCI, and the present study corroborated its association with relationship with Immediate memory. Furthermore, IPL.R is more impaired as MCI progresses to AD (38).

The SN is a large-scale limbic network that co-activates signals required for behavioral change. The SN has been described as a task-positive network that activates corresponding regions during cognitive task performance, and disruption of functional connectivity in the SN has also been suggested to be a significant hallmark of preclinical AD (39). The Rolandic operculum includes sensory, motivational, autonomic, cognitive processing, and language, with impairments primarily related to language deficits (40, 41). Several previous studies have corroborated the attenuation of language functioning in aMCI (42–44), and although naMCI also exhibits impairments in language functioning, aMCI performs worse than naMCI, especially in semantic fluency and language comprehension tasks (3). The above may serve as a reason why aMCI has poorer Rolandic_Oper_L (ROL.L) FC connections than naMCI.

Regarding biomarkers in these brain regions, although PET studies in AD clinical research have yet to yield consistent conclusions due to resolution and sensitivity limitations, they still provide some reference value. In this study, the differentially affected brain areas primarily concentrated in the frontal and parietal lobes. First, reduced glucose metabolism in the parietal and frontal lobes serves as a hallmark of AD (45). Additionally, non-invasive imaging of cerebral amyloid-β serves as one marker for AD. Previous amyloid PET studies have demonstrated that the frontal and parietal lobes in AD also exhibit high binding affinity for amyloid-β (46, 47). These PET studies using various tracers provide limited pathological evidence for AD progression in these brain regions.

Previous evidence suggests that rsn are not independent and have complex interactions (48). weaker FC between DMN, DAN and SN versus stronger FC between SN, SMN suggests balance and regulation between these networks. According to previous studies connectivity barriers between DMN and DAN may be a potential predictor of AD progression (49, 50). In contrast, the connectivity between DMN and SN integrates key networks involved in the detection of internal mental states and associated external stimuli, thereby influencing cognitive processes such as attention, memory retrieval, and decision making (51). Based on previous evidence of DMN-SN network disruption in AD with aMCI (52–54), FC disconnection between the DMN and SN may also be associated with disease progression (55). Furthermore, the reasons for stronger SN-SMN in aMCI were not revealed in previous studies, but SN-SMN was found to show positive correlations with cognitive functioning, especially working memory (56, 57), which is in line with the results of the correlation between MoCA scores and Immediate memory in this paper. The reason for the emergence of stronger in aMCI may be because in the early stages of the pathological process, aMCI patients still have the ability to allocate additional cognitive resources to compensate for varying degrees of cognitive deficits (58). This may imply that these network inhibitions or network imbalances are possible mechanisms by which aMCI is more likely to progress to AD than naMCI.

In addition to FNC changes, DAN-DMN, DMN-SN, and SN-SMN exhibited positive correlations with MoCA, showing their connection to cognition. In addition, we found a positive correlation between DMN-SN connectivity and SN-SMN connectivity and Immediate memory, suggesting that the decoupling between DMN-SN and the enhancement between SN-SMN may be related to the self-regulation of Immediate memory in aMCI.

## 6 Limitations and future directions

Our study has several limitations. First, it is a preliminary cross-sectional comparison of functional connectivity between aMCI and naMCI with a relatively small sample, which limits causal inference about why aMCI cases more frequently convert to Alzheimer’s disease; larger, longitudinal cohorts are required to clarify prognostic relationships. Second, the present analysis considered a restricted set of networks; other systems not included here—such as the cerebellar network, auditory network, and limbic circuitry—might also contribute substantially to the pathophysiology of cognitive decline in aMCI. Third, although ICA is valuable for delineating spatially independent networks, it does not provide information on the directionality of inter-network interactions; future work using methods capable of estimating effective connectivity will be necessary to determine causal influences among networks in aMCI versus naMCI. In addition, conventional resting-state FC captures averaged coupling and is insensitive to rapid temporal fluctuations within network dynamics; therefore, dynamic FNC approaches represent an important avenue for subsequent investigations. Finally, in the absence of pathological or clinical control groups (such as vascular cognitive impairment), we explicitly acknowledge the limitations of inferring disease specificity.

## 7 Conclude

In conclusion, the present study demonstrates that aMCI contrasts naMCI with widespread intra- and inter-static FNC differences, mainly involving the DMN, DAN, SMN, and SN. these network interactions provide a powerful method for assessing and predicting why aMCI is more prone to progressing to AD, and contribute to our understanding of the neurological mechanisms underlying the pathological process of AD.

## Data Availability

The raw data supporting the conclusions of this article will be made available by the authors, without undue reservation.
